# Therapeutic Yoga: A feasible complementary approach for glycemic control in individuals with impaired fasting glucose and elevated HbA1c

**DOI:** 10.1016/j.conctc.2025.101493

**Published:** 2025-05-17

**Authors:** Gitika Bhasin, Rucha S. Dafale, K. Annapoorna, Shobha U. Kamath, Divya Matlani, Raju Rana, Mukhyaprana M. Prabhu, Akhilesh K. Pandey, Sahana Shetty, Lavya Shetty, K. Vasanthalaxmi, S.D. Manjula

**Affiliations:** a Division of Yoga, Centre for Integrative Medicine and Research (CIMR), Manipal, Manipal Academy of Higher Education (MAHE), Manipal, Karnataka, 576104, India; b Department of Physiology, Kasturba Medical College (KMC), Manipal, Manipal Academy of Higher Education (MAHE), Manipal, Karnataka, 576104, India; c Department of Biochemistry, Kasturba Medical College (KMC), Manipal, Manipal Academy of Higher Education (MAHE), Manipal, Karnataka, 576104, India; d Yogananda School of Spirituality and Happiness, Shoolini University of Biotechnology and Management Sciences, Solan District, Himachal Pradesh, 173229, India; e Department of Medicine, Kasturba Medical College (KMC), Manipal, Manipal Academy of Higher Education (MAHE), Manipal, Karnataka, 576104, India; f Department of Community Medicine, Kasturba Medical College (KMC), Manipal, Manipal Academy of Higher Education (MAHE), Manipal, Karnataka, 576104, India; g Department of Endocrinology, Kasturba Medical College (KMC), Manipal, Manipal Academy of Higher Education (MAHE), Manipal, Karnataka, 576104, India

**Keywords:** Impaired fasting glucose, Yoga, Glycemic control

## Abstract

**Background:**

Impaired Fasting Glucose (IFG) with elevated glycated hemoglobin (HbA1c) is a key precursor to type 2 diabetes mellitus (T2DM). Although asymptomatic, IFG significantly raises the risk of developing T2DM and cardiovascular complications, emphasizing the need for early intervention. The Therapeutic Yoga Module (TYM) was designed to offer a feasible and effective remedy for improving glycemic control.

**Methods:**

The TYM was designed by combining different yoga practices including asanas, pranayama, relaxation, and dhyana. After validating the module, a feasibility study was carried out on 12 individuals. Subsequently, the preliminary impact was assessed on 29 individuals (intervention group = 14 and control group = 15) with IFG. The study evaluated practicality, participant acceptance, and changes in Fasting Blood Glucose (FBG) and HbA1c levels over 12 weeks.

**Results:**

The TYM achieved a content validity index (CVI) of 0.75, with 15 out of 20 practices deemed effective by the expert panel. The Intra-Class Correlation (ICC) coefficient of 0.864 indicated strong reliability. Feasibility testing revealed high participant acceptance, with an average attendance rate of 84.9 %. The intervention group showed significant improvements in FBG (from 108.79 mg/dL to 91.00 mg/dL, P < 0.001) and HbA1c (from 6.00 % to 5.73 %, P < 0.001), compared to the control group, which had more modest improvements. The analysis of covariance (ANCOVA) analysis confirmed that these improvements were primarily attributable to TYM.

**Conclusion:**

Preliminary findings suggest that TYM may be a promising complementary intervention for individuals with IFG or at risk of T2DM.

**Trial registration:**

Clinical Trials Registry - India (CTRI); Registration number: CTRI/2022/04/042307; Registration Date: April 29, 2022.

## Highlights

• Impaired Fasting Glucose (IFG) is a major risk factor for Type 2 Diabetes (T2DM).
• Therapeutic Yoga Module (TYM) emerges as a potential preventive therapy for T2DM.
• TYM offers a practical and efficient remedy for glycemic control in IFG.

## 1 Background

Impaired fasting glucose (IFG), a characteristic of prediabetes, represents a state of hyperglycemia characterized by fasting blood glucose (FBG) levels between 100 and 125 mg/dL (5.6–6.9 mmol/L), which are elevated above the normal range yet remain below the threshold for the diagnosis of diabetes mellitus (T2DM) [1]. Similarly, impaired glucose tolerance (IGT) is defined by plasma glucose levels ranging from 140 to 200 mg/dL (7.8–11.0 mmol/L) 2 h after a 75 g oral glucose load [2]. According to the American Diabetes Association (ADA) criteria, both IFG and IGT, along with a glycated hemoglobin (HbA1c) level of 5.7 %–6.4 %, are recognized as indicative of prediabetes [2]. Despite the absence of overt symptoms in most individuals, prediabetes is a significant risk factor for the progression to T2DM, with estimates suggesting that up to 70 % of individuals with prediabetes will eventually develop diabetes [[3], [4], [5]]. The International Diabetes Federation (IDF) projects that by 2035, approximately 471 million individuals globally will have prediabetes [6].

The primary risk factors for prediabetes include obesity, family history of diabetes, hypertension, physical inactivity, dyslipidemia, and polycystic ovarian syndrome [7]. Importantly, prediabetes is not only a precursor to T2DM but also confers an increased risk of cardiovascular disease and stroke [7]. The pathophysiological mechanisms underlying prediabetes parallel those of T2DM, characterized by a compensatory hyperinsulinemic response to hyperglycemia, which, over time, leads to insulin resistance and subsequent metabolic disturbances [8]. This metabolic cascade, if unchecked, can progress to overt diabetes and contribute to the development of metabolic syndrome, with deleterious effects on various organ systems, including the cardiovascular system, retina, kidneys, and peripheral nerves [8].

Seeing the prevalence and lack of awareness among the population, early intervention is crucial in the management of prediabetes to prevent the progression of T2DM and associated complications. Lifestyle modifications, particularly those involving dietary adjustments and increased physical activity, are the cornerstone of prediabetes management [7]. Yoga, a traditional practice rooted in Indian culture and recognized as a form of Complementary and Alternative Medicine (CAM), incorporates elements such as asanas (postures), pranayama (breathing exercises), meditation, and relaxation techniques [9]. Emerging evidence suggests that yoga interventions may benefit glycemic control [10], including improvements in FBG, postprandial glucose levels, HbA1c, and insulin sensitivity [[11], [12], [13], [14], [15], [16]]. Moreover, yoga has been associated with favorable outcomes in anthropometric parameters among individuals with metabolic syndromes [[12], [13], [14]].

Existing yoga protocols such as the Yoga for Type 2 Diabetes Prevention (Yoga-DP) [17] and the Diabetic Yoga Protocol (DYP) [18], also referred to as the Yoga-based Lifestyle Protocol (YLP) [19], have demonstrated efficacy in managing and potentially arresting the progression of T2DM. However, these protocols typically require 60–75 min per session and incorporate components such as prayers and complex practices (e.g., different variations of Surya Namaskar—10-step fast, 12-step slow, chair-based Surya Namaskar; as well as postures requiring greater balance or exertion, such as Sarvangapushti, Parivrtta Trikonasana, Prasarita Padahastasana, Viparita Karani, Ardha Ushtrasana, Agni Sara, and Kapalabhati). While these elements offer potential benefits; complexity and exertion in the postures may present feasibility challenges for middle-aged adults (the most common age group for prediabetes and T2DM development [20,21]), hypertensive individuals, beginners, or those with low physical endurance. Additionally, prayer components in existing protocols may limit inclusivity for individuals from diverse cultural backgrounds, highlighting the need for a more inclusive and adaptable approach. Recognizing these challenges and aligning with ADA recommendations of 150 min of moderate-intensity physical activity per week for individuals at risk of type 2 diabetes [22], we aimed to develop the Therapeutic Yoga Module (TYM) with simple yet effective practices. TYM is designed for a 45-min duration, integrating 25 min of physical postures with relaxation and 20 min of breathing and meditation techniques. Unlike more intense and long protocols that may lead to fatigue or exhaustion, we designed the TYM with simpler and less number of practices with appropriate rest periods between the practices to prevent fatigue and to ensure that participants remain energized and able to manage daily tasks without feeling physically drained. Our objectives were to: i) Design and validate the TYM, ii) Evaluate its feasibility, and iii) Assess its impact on glycemic control in individuals with IFG. The newly developed TYM offers several advantages, including a simplified practice regimen, enhanced feasibility, and adaptability for different age groups across diverse populations while reducing blood glucose levels effectively. Our study seeks to address the unique needs of the prediabetes population, providing a potentially valuable adjunct to conventional management strategies.

## 2 Methods

This study aimed to develop a feasible and effective yoga module for individuals diagnosed with prediabetes who are at a high risk of T2DM. This research followed a quantitative approach, incorporating validation and feasibility assessments and evaluation of glycemic outcomes. Qualitative feedback on feasibility was also collected to supplement the findings. The present study was conducted at multiple sites of Manipal Academy of Higher Education (MAHE), Manipal, Karnataka, India, including Kasturba Hospital (KH), MAHE campus, and Community Centers of Kasturba Medical College (KMC), where participants were screened and recruited; Division of Yoga, Centre for Integrative Medicine and Research (CIMR) and Rural Maternity and Child Welfare (RMCW), Udyavara, Community Center of KMC, where intervention was conducted; Central blood collection lab of KH, where blood samples were collected for pre-post testing of FBG and HbA1c; and Department of Biochemistry and Physiology, KMC, where data analysis was performed. These sites provide access to academic and clinical resources necessary for the successful implementation of the study.

### 2.1 Development and validation of the therapeutic yoga module (TYM)

#### 2.1.1 Designing the therapeutic yoga module (TYM)

The design of the TYM involved a comprehensive review of both classical and contemporary yoga literature. Ancient texts, including *Hatha Yoga Pradipika, Light on Yoga, YogasanagaLu, Asana Pranayama Mudra Bandha,* and *Gheranda Samhita*, were examined to identify relevant yoga practices. Concurrently, recent experimental studies from 2019 onwards, focusing on yoga's therapeutic potential for metabolic syndrome, were reviewed through PubMed and Google Scholar. Keywords such as “Diabetes,” “Impaired Fasting Glucose,” “Yoga,” and “Metabolic Syndrome” were used in conjunction with Boolean operators “AND” and “OR” to refine search results. The selected yoga practices emphasized pancreas stimulation, weight management, adiposity, and common comorbidities like diabetes and hypertension. Consultations with yoga therapy experts from the Division of Yoga, CIMR, MAHE, Manipal further guided the selection process. The finalized TYM included a balanced combination of asanas with conscious breathing, pranayama, bandha, meditation, and relaxation techniques, tailored to the needs of individuals with IFG. The module was standardized to a 45-min duration, and a comprehensive handout was provided to participants for reference and home practice.

#### 2.1.2 Validation of the therapeutic yoga module (TYM)

It was sent to 20 yoga experts with clinical expertise to validate the TYM design, and 11 responded within three months (April 2022 to July 2022). These experts, holding doctorates or master's degrees in yoga and with a minimum of five years of experience, evaluated the module. They rated the usefulness of each yoga practice on a Likert scale of 1–5 (1 - strongly disagree, 5 - strongly agree). The Content Validity Ratio (CVR) for each item was calculated using Lawshe's formula, and items with a CVR of >0.80 (p < 0.05, one-tailed for 11 raters) were retained. Additionally, the Content Validity Index (CVI) and the intra-class correlation (ICC) coefficient for inter-rater reliability were computed to ensure the module's content validity and reliability.

### 2.2 Recruitment and enrollment procedures

Participants were recruited for the single-group feasibility study and the subsequent pilot study through the outpatient departments of Kasturba Hospital (KH), the MAHE campus, and the community health centers of KMC, MAHE. The two studies involved entirely separate participant groups, and no individual participated in both phase of the research.

Interested individuals were screened based on the ADA criteria for prediabetes, specifically FBG levels between 100 and 125 mg/dL and HbA1c levels between 5.7 % and 6.4 %. Those who met the eligibility criteria were provided with a participant information sheet detailing the study procedures.

In the pilot study, individuals who consented to participate were assigned to either the intervention or control group based on their availability and convenience, as randomization was not employed. Participants were required to be adults aged 25–55 years, of any gender with prediabetes, willing to participate in a 12-week study, and without any prior or current regular yoga practice. Individuals were excluded if they had a diagnosed case of T2DM or any other metabolic disorder, severe musculoskeletal, cardiovascular, or neurological conditions that could restrict physical activity, or were on medications that could significantly impact glucose metabolism. Pregnant and lactating women were also excluded from participation.

Participants were required to complete a minimum requirement for sessions and diet adherence to be included in the final analysis, as described in the Statistical Analysis section.

### 2.3 Feasibility testing of the validated Therapeutic Yoga Module (TYM)

The single-group feasibility study of the TYM was tested on 12 individuals included based on eligibility criteria.

To evaluate the TYM's practicality, all 12 participants completed a feedback form assessing the ease of practicing each component of the yoga module. All practices included in the TYM were introduced from the first day of the intervention, allowing participants to experience each practice by the 10th day. The feedback was collected once through a Google Form after the 10th day of the intervention, ensuring participants had sufficient experience to provide meaningful responses. Collecting feedback at this stage also allowed us to assess whether participants could sustain the intervention for the remaining study duration, making it a useful predictor of adherence. Ratings on each practice and overall protocol were collected using a Likert scale ranging from 1 (very difficult) to 5 (very easy) and qualitative feedback was also recorded through an optional open-ended question (“Any additional comments?”) in the feedback form. Confidentiality was maintained, with only age and gender recorded.

Attendance was tracked throughout the 12-week intervention, and both individual attendance rates and the overall average were calculated to assess adherence. This data provided insights into participant engagement and the feasibility of implementing the TYM in real-world settings, which is crucial for its broader application.

### 2.4 Impact of Therapeutic Yoga Module (TYM)

**Fig. 1:**
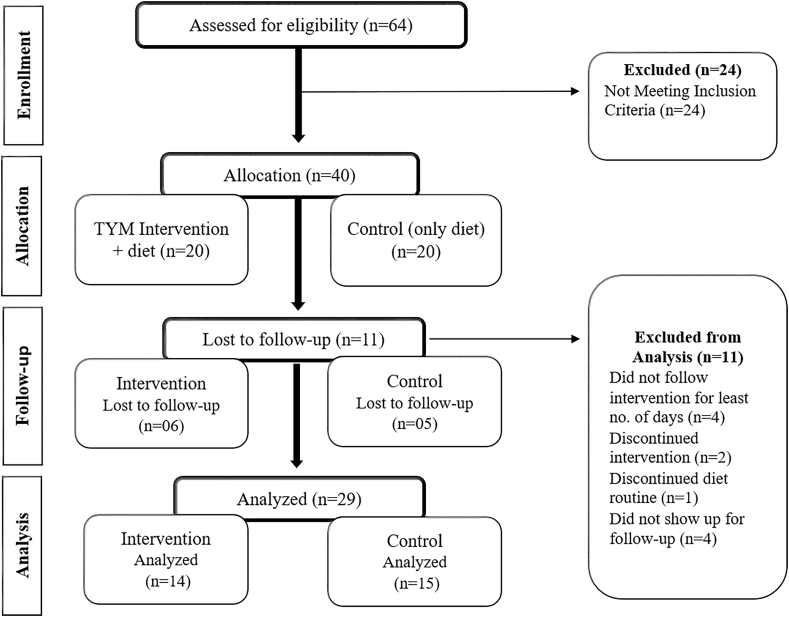
CONSORT flow diagram of participant enrollment, allocation, follow-up, and analysis.

The preliminary impact of the TYM was evaluated by comparing baseline and post-intervention levels of FBG and HbA1c among participants. The study analyzed 29 participants, with 14 in the intervention group who followed the TYM and 15 in the control group who did not undergo the intervention, as shown in Fig. 1.

### 2.5 Intervention

All participants in the feasibility study (TYM group) and the pilot study (TYM and Control group) followed the diet instructions provided by the study physician. The instructions, given verbally, emphasized reducing carbohydrate intake, eliminating added sugars, and incorporating green vegetables into daily meals. These dietary guidelines were intended to complement the intervention and support improvements in blood glucose levels.

The intervention group participated in a structured 12-week yoga program, five days a week. The initial two weeks involved compulsory, supervised sessions led by yoga therapy professionals to ensure correct practice and adaptation. Following this phase, participants had the option to continue home-based sessions for the remaining ten weeks, although active participation in supervised sessions was encouraged throughout the study. All supervised sessions were conducted in groups, each session lasted 45 min, consisting of approximately 25 min of asanas and relaxation, followed by 20 min of breathing techniques and meditation. To support home practice, participants were provided with a TYM instruction booklet, which outlined the sequence of postures and breathing techniques in detail.

Adherence to diet and intervention was monitored through regular telephonic follow-ups conducted once a week throughout the 12-week study period to ensure compliance and provide necessary guidance. During each call, participants were asked to report the number of yoga sessions completed in a week, any missed sessions along with reasons, and whether they followed the dietary recommendations.

Any day participants deviated from the recommended diet (e.g., consumption of restricted items such as sugar, sweets, high-glycemic fruits, or rice) was recorded as a day of non-compliance. Similarly, any week during which participants completed fewer than three yoga sessions (either supervised or home-based) was considered indicative of reduced intervention adherence.

### 2.6 Statistical Analysis

Descriptive statistics were employed to summarize the feedback form data, including the percentage of participants who rated each yoga practice as easy to perform (ratings of 4 or 5) versus those who found it difficult (ratings below 4). The overall ease of TYM was also evaluated using the same rating scale. Additionally, attendance rates and diet adherence were computed as percentages, both for individual participants and as a group average, to assess adherence to the intervention.

A Per-Protocol (PP) analysis was conducted to evaluate the impact of the TYM on glycemic control. Participants were included in the final analysis only if they met the minimum adherence criteria. Both groups were required to follow the diet instructions for more than 80 % of days (68 out of 84 days) throughout the study. Additionally, participants in the intervention group were required to attend at least 60 % (36 out of 60) of the sessions. Participants who were lost to follow-up, discontinued participation, or had incomplete post-intervention data were excluded from the final analysis. Blood samples were not collected from those who did not meet the minimum criteria, as their exposure to the intervention or diet was insufficient for a meaningful comparison with other participants in the study. However, their attendance and diet adherence data were recorded to provide a more comprehensive feasibility assessment.

For the primary outcome measures, FBG and HbA1c levels, mean and standard deviation (SD) values were calculated to describe the distribution and variability of these biomarkers across the study period.

To evaluate the impact of the TYM, we employed several statistical methods. Intragroup analysis was conducted using paired t-tests to compare pre- and post-intervention levels of FBG and HbA1c within the intervention group, assessing changes attributable to the TYM. To determine the relative effectiveness of the TYM, independent t-tests were used to compare post-intervention FBG and HbA1c levels between the intervention group and the control group, thus evaluating whether the TYM had a significantly greater effect on glycemic control compared to diet alone. Additionally, to ensure the robustness of our findings, we performed the analysis of covariance (ANCOVA) to adjust for baseline values and adherence to the prescribed diet. Statistical significance was set at a p-value of <0.05, and all analyses were conducted using appropriate statistical software to ensure the accuracy and reliability of the results.

## 3 Results

### 3.1 Content validation

**Fig. 2:**
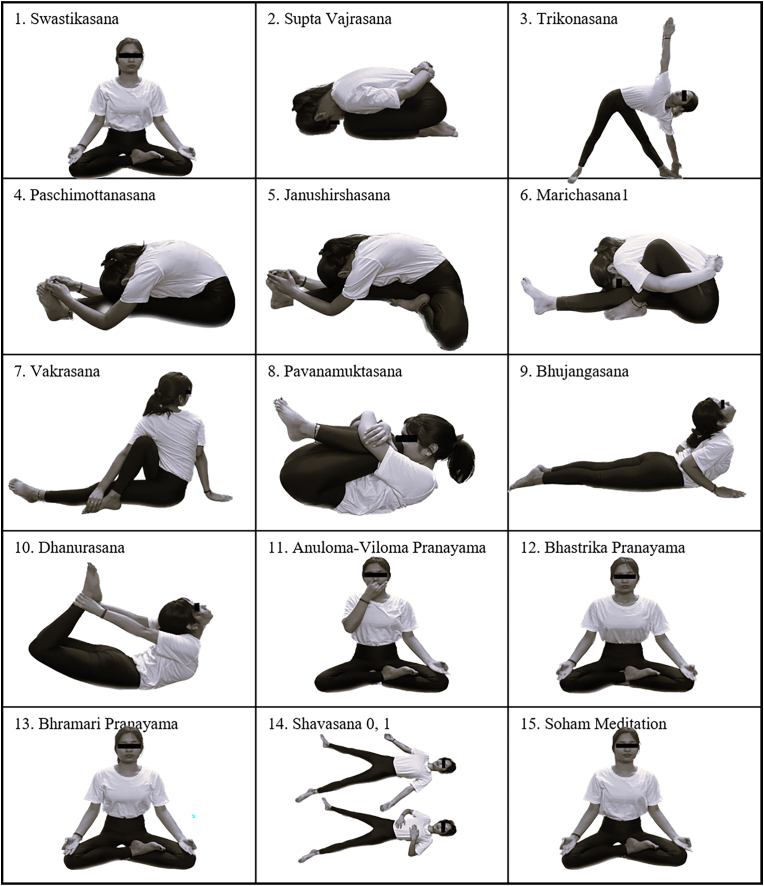
Finalized Therapeutic Yoga Module (TYM) after content validation.

Eleven experts specializing in yoga therapy and research agreed to validate the TYM's content. Supplementary Table 1 displays the expert ratings for individual practices and the calculated CVR using Lawshe's formula. 15 of the 20 practices that were sent for validation had CVR values above 0.80 and were therefore added to the final module as shown in Fig. 2. The overall module's CVI of 0.75 suggests 75 % agreement among experts, representing good content validity for the TYM. Inter-rater reliability has an ICC of 0.864 and a 95 % confidence interval of 0.712–0.956.

### 3.2 Feasibility testing

In the feasibility study, 12 individuals participated, including 7 females (58.33 %) and 5 males (41.66 %), with an average age of approximately 45 years. Their baseline glucose levels averaged 108.3 mg/dL, with an average HbA1c level of 6.03 %.

#### 3.2.1 Participants’ feedback on Therapeutic Yoga Module (TYM)

Quantitative feedback: The feedback on the ease of practice was quantified through a percentage breakdown of participants' ratings for each practice and the overall protocol, as shown in Supplementary Table 2. The average overall protocol rating reported was 4, indicating a high level of satisfaction and perceived ease of practice. Additionally, it is noteworthy that all participants rated the overall protocol with a score of >3, further indicating positive perceptions of the protocol's accessibility and usability.

Qualitative feedback: The participants provided overwhelmingly positive feedback on the ease of practice and the overall experience with the yoga protocol. Many expressed feeling calm, relaxed, and active after the yoga sessions, with noticeable improvements in sleep quality. Some participants initially found certain practices challenging but reported significant progress and increased ease with consistent practice. The instruction booklet was praised for its clarity and effectiveness in facilitating self-practice. Overall, participants found the yoga protocol accessible, especially for new practitioners, and noted significant improvements in well-being and mood throughout the intervention.

#### 3.2.2 Participants’ attendance rate

The average attendance rate for the participants was 84.9 %. Each participant had more than 75 % attendance, with all attendees engaging in either supervised sessions or home practice for at least three days per week throughout the 12-week interventional duration.

### 3.3 Pilot study

A total of 40 participants (20 in each group) consented and were initially included in the pilot study. However, 11 participants (6 from the intervention group and 5 from the control group) were excluded from the pre-post glycemic outcome analysis due to loss to follow-up, withdrawal, or insufficient adherence, resulting in 29 participants completing the study. In the intervention group, 2 participants discontinued the practice midway through the study, and all 6 failed to meet the minimum session requirement. In the control group, 1 participant stopped following dietary instructions after the third week, while 4 did not attend the post-assessment.

#### 3.3.1 Demographic characteristics

The intervention group (n = 14) had a mean age of 43.1 ± 8.19 years and included 6 (42.85 %) males and 8 (57.15 %) females. The control group (n = 15) had a mean age of 39.5 ± 8.49 years, with 6 (40 %) males and 9 (60 %) females. The average height and weight were 168 ± 9.16 cm and 68.9 ± 16.2 kg in the intervention group and 164 ± 9.07 cm and 72.7 ± 8.26 kg in the control group. Most participants in both groups had completed undergraduate or postgraduate education and were working. There were no significant baseline differences between groups.

#### 3.3.2 Adherence rate

Among the 29 participants who completed the study, the intervention group (n = 14) had an average attendance rate of 85.6 %, while diet adherence in the intervention and control groups was 91.3 % and 91 %, respectively, indicating strong compliance with dietary recommendations. In contrast, participants who did not complete the intervention (n = 6) had a lower attendance rate of 46.4 %. The primary reasons for dropout included work schedules, night shifts, personal commitments, and reduced interest, rather than difficulty in performing yoga practices. These reasons were recorded during follow-up calls and documented when participants informed the researcher of their decision to discontinue, allowing us to assess the nature of non-adherence.

### 3.4 Changes in key biomarkers

#### 3.4.1 Intragroup comparison (shown in Table 1)

##### 3.4.1.1 Intervention group

**Fig. 3:**
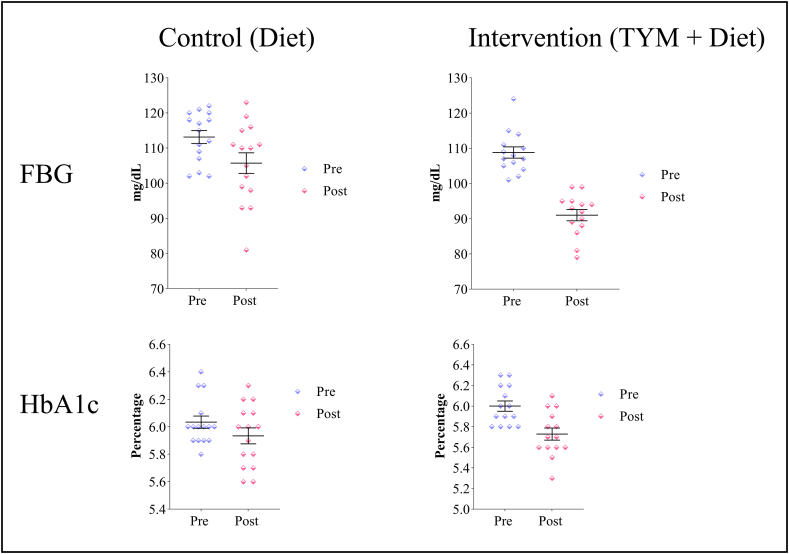
Comparison of pre-and post-intervention fasting blood glucose (FBG) and HbA1c levels between the control (diet only) and intervention (TYM + diet) groups.

The participants in the TYM intervention group (n = 14) demonstrated significant improvements in both FBG and HbA1c levels after the 12-week intervention, as shown in Fig. 3. The mean FBG level decreased from 108.79 mg/dL (±5.964) at baseline to 91.00 mg/dL (±5.897) post-intervention, a reduction that was statistically significant with a t-value of 7.67 and a p-value of <0.001, providing preliminary support for the beneficial effects of the TYM on glycemic control. Similarly, the mean HbA1c level showed a significant reduction, decreasing from 6.00 % (±0.188) to 5.73 % (±0.220) after the intervention. This reduction was also statistically significant, with a t-value of 10.21 and a p-value of <0.001, further indicating the potential of the TYM in improving glycemic outcomes.

##### 3.4.1.2 Control group

The control group (n = 15), which did not participate in the TYM but adhered to the same diet, showed modest improvements in glycemic markers, as shown in Fig. 3. The mean FBG level decreased from 113.13 mg/dL (±7.130) at baseline to 105.73 mg/dL (±11.380) after 12 weeks. This reduction, though statistically significant with a t-value of 3.23 and a p-value of 0.006, was less pronounced compared to the intervention group. Similarly, the mean HbA1c level decreased from 6.03 % (±0.172) to 5.93 % (±0.226), with a t-value of 2.29 and a p-value of 0.038. While this change was also significant, the magnitude of the reduction was smaller than that observed in the intervention group.

#### 3.4.2 Intergroup comparison

##### 3.4.2.1 Post comparison between groups shown in Table 2

The post-comparison between groups revealed that the intervention group had significantly greater reductions in FBG and HbA1c than the control group (t-value = 4.315, p < 0.001 and t-value = 2.472, p = 0.020, respectively).

##### 3.4.2.2 ANCOVA analysis

To account for any potential baseline differences and diet adherence, an ANCOVA was performed, as shown in Table 3. The analysis revealed that the TYM intervention had a significant independent effect on glycemic outcomes, even after adjusting for diet adherence. The intervention group showed a significantly greater reduction in both FBG (F = 21.10, p < 0.001) and HbA1c (F = 13.63, p = 0.001) compared to the control group. Although diet adherence also had a significant impact (FBG: F = 10.17, p = 0.004; HbA1c: F = 7.38, p = 0.012), the higher F-values for the intervention suggest that the improvements in glycemic outcomes were primarily driven by TYM, rather than diet alone.

**Table 1:** Changes in Biomarkers after 12 weeks in the Intervention (yoga) group and Control group.

Variable	group	N	Timepoints		t-value	p-value∗
			Pre (Mean ± SD)	Post (Mean ± SD)		
**FBG (mg/dL)**	Yoga	14	108.79 ± 5.964	91.00 ± 5.897	7.67	<0.001
	control	15	113.13 ± 7.130	105.73 ± 11.380	3.23	0.006
**HbA1c (%)**	Yoga	14	6.00 ± 0.188	5.73 ± 0.220	10.21	<0.001
	control	15	6.03 ± 0.172	5.93 ± 0.226	2.29	0.038

**Table 2:** Post comparison between groups.

Variable	Groups		t-value	p-value∗
	Yoga (Mean ± SD)	Control (Mean ± SD)		
**Post FBG (mg/dL)**	91.00 ± 5.897	105.73 ± 11.380	4.315	<0.001
**Post HbA1c (%)**	5.73 ± 0.220	5.93 ± 0.226	2.472	0.020

**Table 3:** ANCOVA- post FBG and post HbA1c.

Variables	Source	F-value	p-value
**Post FBG**	Group	21.10	<0.001
	Pre FBG	3.40	0.077
	Adherence to diet	10.17	0.004
**Post HbA1c**	Group	13.63	0.001
	Pre HbA1c	52.51	<0.001
	Adherence to diet	7.38	0.012

## 4 Discussion

Yoga, an ancient science, has emerged in the modern world with its holistic therapeutic approach to promote overall well-being and enhance the quality of life. The holistic approach of yoga combines various practices to manage several health conditions [23]. With the increasing interest in the role of yoga therapy in managing different health conditions, the present study projected to design an accessible and effective therapeutic yoga module specifically for individuals with IFG and elevated HbA1c levels. Existing yoga protocols in this background include a variety of practices such as Shithilikarana Vyayama (loosening exercises), Surya Namaskar (sun salutation exercises), Asana (yogic poses), Pranayama (breathing practices), Dhyana (meditation), and relaxation techniques [[17], [18], [19]]. The DYP/YLP protocols also incorporate prayers and kriya (cleansing practices) [18,19]. Incorporating these components into the module may limit inclusivity, particularly for participants from diverse cultural backgrounds, while complex and exhaustive practices may pose challenges for those with common age-related or prediabetes-associated health issues such as hypertension, joint stiffness, or reduced mobility.

Considering these factors, the TYM for prediabetes management was meticulously designed after a thorough review of the literature. Recognizing the different religious backgrounds, our module excludes prayer components, ensuring it is accessible to participants from diverse religious backgrounds and enhances inclusivity. Instead, the module starts with Swastikasana (auspicious pose) and conscious breaths, establishing a welcoming and inclusive environment for all. Swastikasana is an ideal meditative posture and a preferable position for practicing pranayama and meditation [9]. Contrasting previous protocols, the TYM does not include complex or exhaustive practices such as Sarvang Pushti, Parvritta trikonasana, Prasarita Padhastasana, Surya Namaskar, etc. which reduces the risk of injury and improves adherence among the different age groups. During the designing phase, our module included a total of 20 yoga practices with 13 Physical postures (Asanas), 4 breathing techniques (Pranayamas), 1 lock (Uddiyana Bandha), 1 relaxation technique (Shavasana), and 1 Meditation (Soham Dhyana). The module's 45-min duration and simplicity are optimized for regular practice, making it appropriate for beginners and those with lower physical stamina. The inclusion of 25 min of physical activity per session followed by breathing and meditation techniques in the TYM also aligns with the ADA recommendation of physical activity for individuals at risk of developing T2DM [22].

The designed TYM was then validated by consulting with yoga professionals from different universities and institutes in India. The experts agreed that the developed module's content was useful for stimulating the pancreas, lowering blood glucose levels, losing weight, managing stress, and enhancing the quality of life in prediabetic patients. Most of the practices are well known for their intense capability to directly affect pancreatic stimulation [24]. Based on two factors, we modified the module: i) The practices that received ratings of 4 and 5 from more than 90 % of the panelists were considered as having a content validity ratio of 0.80 and above; ii) the suggestions and comments from the experts were incorporated. Ultimately, 15 out of 20 items from the initial compilation were retained in the final TYM.

During the validation process, the following practices scored lower than the cut-off values (rated CVR less than 0.80): Vajrasana, Purvottanasana, Jathara Parivartanasana, Suryabhedana, and Uddiyana Bandha. The rest of the practices were marked as useful by more than 90 % of the experts and retained in the final TYM. The experts particularly endorsed the forward bending, backward bending, and twisting postures for their ability to improve circulation and adipose reduction, mainly in the waistline area, and stimulate the secretions of the abdominal organs [25,26]. Pranayama techniques were also highly valued for balancing the nervous system, calming the mind, and aiding stress reduction while relaxation techniques and Soham meditation were recognized for their role in stimulating the parasympathetic system [27,28].

The first feasibility testing of the TYM provided insightful feedback ratings after 10 days of practice on the module's accessibility and ease of practice based on the Likert Scale [[1], [2], [3], [4], [5]]. Slight difficulty in a few practices was observed in the first few days of practice, by the second week, noticeable improvement and better clarity in performing the steps were observed, indicating sufficient familiarity with the practices. Although formal feedback was collected on Day 10, participant progress and continued improvement were observed and self-reported during follow-up calls, maintaining positive trends throughout the intervention. Participants' high attendance rates of more than 75 % and positive feedback highlight the practical implementation of the TYM in real-world settings. Participants reported that the overall module was easy to practice, improving their overall well-being. This aligns with findings from previous studies that underscore the importance of adherence to intervention for good outcomes [29]. Our protocol's high adherence is particularly significant, as increased adherence can amplify the intervention's effects by 26 %, emphasizing the importance of strategies to maximize adherence, especially in interventions requiring more effort than simple pill-taking [29,30].

The qualitative feedback suggests that the TYM effectively addressed common barriers to regular physical activity, such as complexity in the practices, maintaining regular engagement, and challenges related to self-practice [31,32]. Participants felt more relaxed and active, and experienced improvements in sleep quality, which is crucial for glycemic control [33]. The participants' positive experiences with the module resonate with its simplicity, ease of practice, and higher adherence which is linked with better health outcomes [29].

In physical postures, the duration of each posture was synchronized with one's breath, typically held for five breaths to optimize benefits, and focus was placed on mindful breathing throughout the practice [9,34]. Adequate rest periods, ranging from a few seconds to a minute, were provided between practices to promote recovery and improve performance. This approach aligns with the early-stage nature of prediabetes, which typically requires moderate-intensity physical activity and can reduce the risk of T2DM [35]. The ability to complete the practice within a manageable time frame likely contributed to the high adherence rates observed.

A significant aspect of the study assessed the impact of the TYM on glycemic control by comparing the intervention group to a control group. The TYM group showed notable reductions in both FBG and HbA1c levels after 12 weeks, with FBG decreasing to 91.00 mg/dL, bringing it within the normal range, and HbA1c reducing to 5.73 %, nearing normal glycemic levels. This reduction is clinically significant, as maintaining lower FBG levels is crucial for preventing the progression from prediabetes to T2DM and for long-term health benefits [36]. Similarly, the reduction in HbA1c levels in the intervention group highlights improved glycemic control. HbA1c reflects average blood glucose levels over the past two to three months [37] and even a small reduction of 0.2 % in HbA1c levels could lower the risk of diabetes-related complications and mortality by 10 % [38]. In contrast, while the control group showed statistically significant reductions, average FBG and HbA1c levels remained in the prediabetic range (105.73 mg/dL and 5.93 %, respectively). These findings highlight the superior effectiveness of TYM over dietary modifications alone in restoring normal blood glucose levels.

To confirm that these improvements were attributable to the TYM intervention, an ANCOVA was performed, adjusting for baseline values and diet adherence. The analysis demonstrated that the TYM group had significantly greater reductions in both FBG (F = 21.10, p < 0.001) and HbA1c (F = 13.63, p = 0.001), reinforcing the intervention's effectiveness independent of diet adherence.

The observed improvements in glycemic control align with existing literature that supports the effectiveness of yoga in managing blood glucose levels. Yoga practices, through a combination of physical postures, breathing exercises, and relaxation techniques, enhance insulin sensitivity, improve glucose uptake, and reduce stress, which collectively contributes to better glycemic control [39,40].

The high adherence rates, combined with the positive feedback ratings on the module's ease of practice, underscore the feasibility and acceptability of the TYM for individuals with prediabetes. This suggests that the TYM is not only effective in improving glycemic control but also practical and engaging for participants. One of the strengths of the TYM lies in its simplicity and inclusivity, making it accessible to individuals across different age groups, diverse populations, beginners, and those with low physical endurance. The module was designed with limited yet effective practices, an optimal 45-min duration, and a structure that reduces the risk of injury while ensuring feasibility and adherence. By keeping the components simple and adaptable, the TYM enhances acceptability while maintaining its therapeutic effectiveness. However, certain limitations should be acknowledged. The study utilized a small sample size (n = 29), making it a preliminary exploration, and larger studies are needed to improve generalizability. Additionally, the absence of an active control group prevents direct comparisons between TYM and other structured physical activity interventions, although both groups followed standard dietary recommendations.

Another limitation is the lack of randomization in group allocation, which may introduce selection bias. Additionally, while ANCOVA was used to adjust for baseline values and diet adherence, other potential confounding factors such as variations in physical activity outside the study, sleep quality, or stress were not controlled for and should be considered in future research. Furthermore, the study followed a PP approach, which ensures the true effects of the intervention if it is followed correctly, but it may limit real-world applicability compared to an Intention-to-Treat (ITT) analysis that accounts for all enrolled participants regardless of adherence.

Despite these limitations, the study contributes to existing literature by addressing gaps in yoga-based interventions for prediabetes and metabolic syndromes. While the TYM is designed to be safe and inclusive, individuals with certain comorbidities such as asthma, autoimmune disorders, HIV, chronic inflammatory diseases, coronary artery disease, psychiatric disorders, or acute conditions like pregnancy should be mindful of potential contraindications before engaging in yoga practice. Future research could explore ways to further enhance the inclusivity of the module while incorporating a broader range of metabolic and other associated markers to gain deeper insights into its effects.

By recognizing these areas, the study sets the foundation for more extensive research to validate the TYM's impact on managing prediabetes and other metabolic syndromes.

## 5 Conclusion

The TYM developed in this study significantly improved FBG and HbA1c levels in individuals with IFG over 12 weeks, outperforming the control group that followed dietary recommendations alone. The high adherence rates and positive participant feedback highlight the module's feasibility, accessibility, and effectiveness as a non-pharmacological intervention for managing prediabetes. The ANCOVA analysis confirmed that the improvements in glycemic control were primarily due to TYM, independent of diet adherence. These findings suggest that TYM, as a complementary and alternative treatment, could be a valuable tool in preventing the progression of T2DM. However, further research is needed to confirm its long-term efficacy and applicability to a broader population.

## CRediT authorship contribution statement

**Gitika Bhasin:** Writing – original draft, Software, Project administration, Formal analysis, Data curation. **Rucha S. Dafale:** Visualization, Data curation. **Annapoorna K.:** Methodology, Conceptualization. **Shobha U. Kamath:** Validation, Resources, Investigation. **Divya Matlani:** Writing – review & editing. **Raju Rana:** Data curation. **Mukhyaprana M. Prabhu:** Methodology, Investigation. **Akhilesh K. Pandey:** Validation, Formal analysis. **Sahana Shetty:** Methodology. **Lavya Shetty:** Methodology. **Vasanthalaxmi K.:** Conceptualization. **Manjula S.D.:** Writing – review & editing, Supervision, Methodology.

## Ethics approval and consent to participate

The study was approved by the Institutional Ethical Committee of Kasturba Medical College and KH, Manipal, Karnataka, India (723/2021). Informed consent was obtained from all individual participants included in the study. Participants received a detailed information sheet explaining the study's purpose, procedures, potential risks, and benefits. They were allowed to ask questions and were assured that their participation was voluntary and that they could withdraw from the study without any negative consequences.

## Consent for publication

Not applicable.

## Availability of data and materials

Data will be made available upon request from the corresponding author.

## Funding

This study received support from the contingency funds provided by the 10.13039/100019304Manipal Academy of Higher Education (MAHE), Manipal, Karnataka-576104, India, and the 10.13039/501100001501University Grants Commission (UGC), India.

## Declaration of competing interest

The authors declare that they have no known competing financial interests or personal relationships that could have appeared to influence the work reported in this paper.

## Data Availability

Data will be made available on request.

## List of Abbreviations

ADA: American Diabetes Association
ANCOVA: Analysis of covariance
CAM: Complementary and Alternative Medicine
CIMR: Centre for Integrative Medicine and Research
CVI: Content Validity Index
CVR: Content Validity Ratio
DYP: Diabetic Yoga Protocol
FBG: Fasting Blood Glucose
HbA1c: Glycated Hemoglobin
ICC: Intra-Class Correlation
IDF: International Diabetes Federation
IFG: Impaired Fasting Glucose
IGT: Impaired Glucose Tolerance
ITT: Intention-to-Treat
KH: Kasturba Hospital
KMC: Kasturba Medical College
MAHE: Manipal Academy of Higher Education
PP: Per-Protocol
SD: Standard Deviation
T2DM: Type 2 Diabetes Mellitus
TYM: Therapeutic Yoga Module
YLP: Yoga-based Lifestyle Protocol
Yoga-DP: Yoga for Type 2 Diabetes Prevention
