# Supplementary material for: Therapeutic Yoga: A feasible complementary approach for glycemic control in individuals with impaired fasting glucose and elevated HbA1c
Source: Contemp Clin Trials Commun. 2025 May 17;45:101493. doi: 10.1016/j.conctc.2025.101493 (PMC12148726; doi:10.1016/j.conctc.2025.101493)
Supplement: Multimedia component 1 [file mmc1.docx]

**Supplementary Table 1.** TYM validation from experts for patients with prediabetes.

| Yoga practices | 1* | 2* | 3* | 4* | 5* | Experts agree (rating >3) | % of agreement | | CVR* | Remarks |
| --- | --- | --- | --- | --- | --- | --- | --- | --- | --- | --- |
| Swastikasana | 0 | 0 | 1 | 6 | 4 | 10 | 90.9 | | 0.81 | Retained |
| Vajrasana | 0 | 1 | 3 | 4 | 3 | 7 | 63.6 | | 0.27 | Deleted |
| SuptaVajrasana | 0 | 0 | 1 | 3 | 7 | 10 | 90.9 | | 0.81 | Retained |
| Trikonasana | 0 | 0 | 0 | 3 | 8 | 11 | 100 | | 1 | Retained |
| Paschimottanasana | 0 | 0 | 0 | 2 | 9 | 11 | 100 | | 1 | Retained |
| Purvottanasana | 0 | 1 | 2 | 2 | 6 | 8 | 72.7 | | 0.45 | Deleted |
| Janushirshasana | 0 | 0 | 0 | 2 | 9 | 11 | 100 | | 1 | Retained |
| Marichasana1 | 0 | 1 | 0 | 4 | 6 | 10 | 90.9 | | 0.81 | Retained |
| Vakrasana | 0 | 0 | 1 | 4 | 6 | 10 | 90.9 | | 0.81 | Retained |
| Pavanamuktasana | 0 | 1 | 0 | 2 | 8 | 10 | 90.9 | | 0.81 | Retained |
| Bhujangasana | 0 | 0 | 1 | 3 | 7 | 10 | 90.9 | | 0.81 | Retained |
| Dhanurasana | 0 | 0 | 1 | 5 | 5 | 10 | 90.9 | | 0.81 | Retained |
| Jathara Parivartanasana | 0 | 0 | 3 | 3 | 5 | 8 | 72.7 | | 0.45 | Deleted |
| Anuloma Viloma | 0 | 0 | 1 | 4 | 6 | 10 | 90.9 | | 0.81 | Retained |
| Suryabhedana | 0 | 1 | 2 | 5 | 3 | 8 | 72.7 | | 0.45 | Deleted |
| Bhastrika | 0 | 0 | 1 | 3 | 7 | 10 | 90.9 | | 0.81 | Retained |
| Bhramari | 0 | 0 | 1 | 3 | 7 | 10 | 90.9 | | 0.81 | Retained |
| Uddiyana Bandha | 0 | 2 | 0 | 4 | 5 | 9 | 81.8 | | 0.63 | Deleted |
| Shavasana | 0 | 1 | 0 | 1 | 9 | 10 | 90.9 | | 0.81 | Retained |
| Soham Meditation | 0 | 1 | 0 | 4 | 6 | 10 | 90.9 | | 0.81 | Retained |
| Content validation index (CVI) | | | | | | |  | **0.75** | | |
| Intraclass correlation (ICC) coefficient | | | | | | |  | **0.864 (0.712 – 0.956)** | | |

*CVR- content validation ratio. 1- strongly disagree, 2- disagree, 3- neither agree nor disagree, 4- agree, and 5- strongly agree.

**Supplementary Table 2.** Participants’ rating on each practice and overall protocol.

| **Yoga practices** | **participants’ rating**  **(≥ 4)** | **% of ease in practice** | **participants' rating**  **(< 4)** | **% of difficulty in practice** |
| --- | --- | --- | --- | --- |
| Swastikasana | 11 | 91.67 | 1 | 8.33 |
| Supta Vajrasana | 10 | 83.33 | 2 | 16.67 |
| Trikonasana | 12 | 100 | 0 | 0 |
| Paschimottanasana | 8 | 66.67 | 4 | 33.33 |
| Janushirshasana | 10 | 83.33 | 2 | 16.67 |
| Marichasana1 | 6 | 50 | 6 | 50 |
| Vakrasana | 10 | 83.33 | 2 | 16.67 |
| Pavanamuktasana | 11 | 91.67 | 1 | 8.33 |
| Bhujangasana | 12 | 100 | 0 | 0 |
| Dhanurasana | 9 | 75 | 3 | 25 |
| Anuloma Viloma | 12 | 100 | 0 | 0 |
| Bhastrika | 11 | 91.67 | 1 | 8.33 |
| Bhramari | 12 | 100 | 0 | 0 |
| Shavasana | 11 | 91.67 | 1 | 8.33 |
| Soham Meditation | 12 | 100 | 0 | 0 |
| **Overall Protocol** | **12** | **100** | **0** | **0** |
