# Supplementary material for: Therapeutic Yoga: A feasible complementary approach for glycemic control in individuals with impaired fasting glucose and elevated HbA1c
Source: Contemp Clin Trials Commun. 2025 May 17;45:101493. doi: 10.1016/j.conctc.2025.101493 (PMC12148726; doi:10.1016/j.conctc.2025.101493)

Therapeutic Yoga Module for Prediabetes

<u>Instruction Booklet</u>

S. No.	Yoga Practices	Duration (in mins)
**1.**	**Asanas (Physical postures)**<br><u>Meditative posture:</u><br>Swastikasana<br><u>Forward-bending postures:</u><br>Supta-Vajrasana, Paschimottanasana, Janushirshasana, Marichasana1, Pavanamuktasana<br><u>Side bending & twisting postures:</u><br>Trikonasana, Vakrasana<br><u>Backward bending postures:</u><br>Bhujangasana, Dhanurasana	20
**2.**	**Pranayama (Breathing techniques)**<br>Anuloma-Viloma, Bhastrika, Bhramari	10
**3.**	**Shithilikaran (Relaxation technique)**<br>Shavasana 0, Shavasana 1	5
**4.**	**Dhyana (Meditation)**<br>Soham Meditation	10
**Total Duration (in mins)**		**45**

Indications:

TYM is designed for individuals with prediabetes and can be practiced by individuals who are at risk of type 2 diabetes.

Contraindications:

People suffering from acute diseases or serious health conditions should avoid practicing without any guidance.

Pregnant women should avoid practicing this protocol

Steps with Instructions

1. **Swastikasana**:

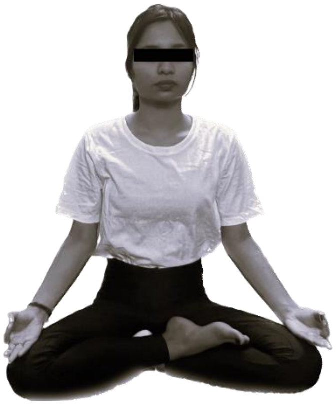

Step I: Sit on the floor with legs stretched out, and palms on the ground. Breathe for 10 times.
Step II: While exhaling fold the left leg and keep its sole touching to the inner side of the right thigh. Inhale deeply.
Step III: While exhaling fold the right leg and keep it between the left thigh and calf of the left leg.
Step IV: Sit erectly and place your hands in chin mudra*.
Step V: Breathe approximately for 1 minute.
Step VI: Unfold the right leg while inhaling. Again inhaling unfold the left leg.

**for chin mudra- join the tip of your thumb and index finger*

2. **Supta Vajrasana:**

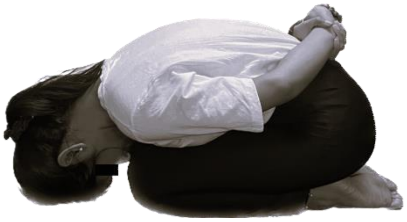

Step I: Sit in Vajrasana (while exhaling fold your legs and sit on your heels).
Step II: Take your hands back, hold the wrist of one hand, and inhale there.
Step III: Exhaling bend forward and touch the ground by your head. Breathe 5 times.
Step IV: Inhaling come up in Vajrasana position and unfold your legs.

3. **Trikonasana**:

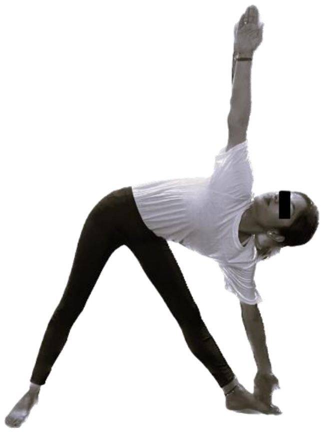

Step I: Stand erect. Inhaling spread the right leg to one leg distance and keep the hands at sideways shoulder level.
Step II: While exhaling turn your right foot to the right side, bend to the right side, and hold the right big toe. Look up. Breathe 5 times.
Step III: while inhaling come up to step I.
Step IV: Exhaling turn your left foot to the left side, bend to the left side, and hold the left big toe. Look up. Breathe 5 times.
Step V: Inhaling come up to step I. While exhaling, join the right leg to the left.

4. **Paschimatanasana**:

   Step I: sit on the ground with your legs stretched out. Slowly inhale.

   Step II: While exhaling bend forward and try to hold your big toes. Breathe 5 times. Breathe in, come up, and release the hands.

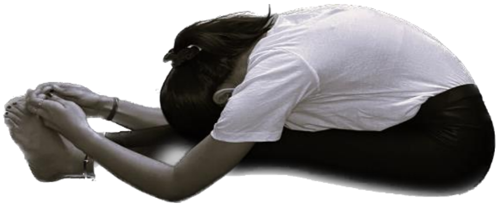

5. **Janushirshasana**:

   Step I: Sit on the ground with stretched legs.

   Step II: Exhaling fold the left leg and keep the heel in such a way that it should touch the perineum. Inhale there itself.

   Step III: While exhaling, bend forward, hold the right big toe, and touch your forehead to the knee. Breathe 5 times.

   Step IV: While inhaling, come up, release the hands, and exhale there.

   Step V: Inhaling spread the left leg.

   Repeat all the procedures on the other side.

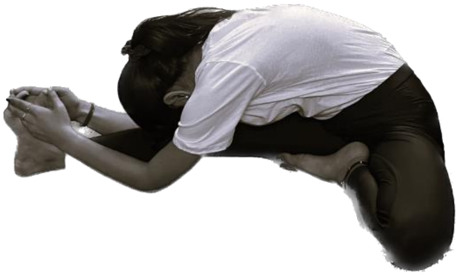

6. **Marichasana 1**:

   Step I: Sit on the floor with stretched legs.

   Step II: Inhaling fold the left leg, knee facing upward direction.

   Step III: Exhaling bring the left hand forward and wrap it around the left shin. Hold the left hand by the right hand on the back.

   Step IV: Exhaling bend forward, touch your forehead to the knee, and breathe 5 times.

   Step V: Inhaling release the hands and come up.

   Step VI: Exhaling spread the leg.

   Repeat the same on the other side.

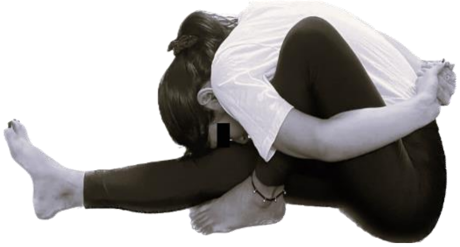

7. **Vakrasana:**

   Step I: Sit on the floor with stretched legs.

   Step II: Inhaling fold the right leg, knee facing upwardly.

   Exhaling, keep the right foot on the left side beside the left knee.

   Step III: While exhaling bring the left hand over the right knee and hold the right big toe. Keep the right hand on the floor (backside). Turn back to the right side. Breathe 5 times.

   Step IV: Inhaling release the hands and turn to the front. Exhale there.

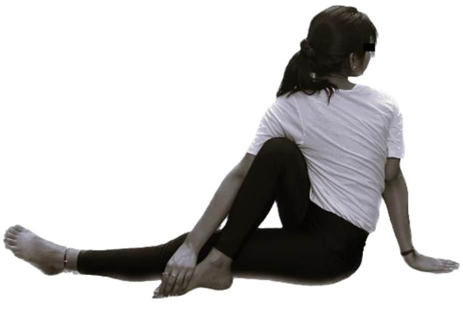

Step V: Inhaling keep the right foot on the right side and exhaling spread it.
Repeat the same to the left side also.

8. Pavanamuktasana:

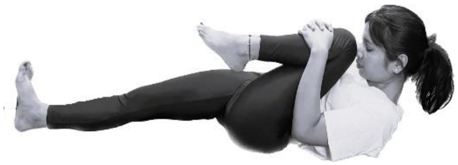

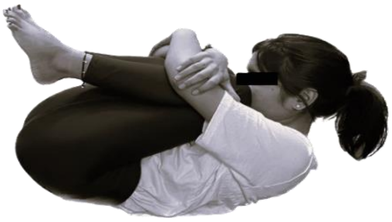

Lie down flatly on your back.
Step I: While exhaling fold your right leg and hold it tightly with both hands.
Step II: Inhaling raise your head and try to touch your right knee. Breathe 5 times.
Step III: Exhaling keep your head down.
Step IV: Inhaling unfold your right leg.
Repeat the same procedure with the left leg and both legs.

9. Bhujangasana:

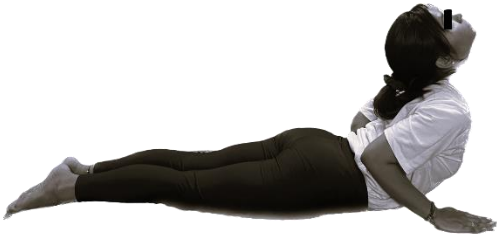

Lie down on your stomach and keep your palms below the head. Relax for 10 breathings. Then keep your palms below your shoulders.
Step I: Inhaling lift your head, chest, and stomach by stretching the back. Look up. Breathe 5 times.
Step II: Exhaling, slowly come down.

10. Dhanurasana:

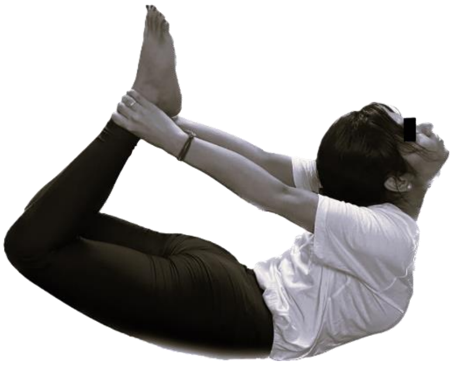

Step I: Lie down on your stomach.
Step II: Fold the legs while exhaling, with inhalation hold the ankles by respective hands and raise your head. Look up.
Breathe 5 times.
Step III: Exhaling release your hands and keep your head on the ground.
Step IV: Inhaling spread the legs.

Preparation for pranayama

Sit in Swastikasana with spine erect, take a few deep breaths.

11. Anuloma-Viloma Pranayama:

Hold Pranav Mudra* from your right hand.

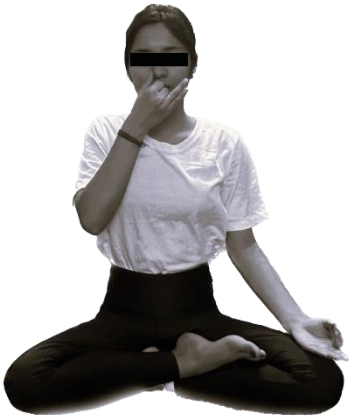

Close your right nostril and inhale from the left nostril. After complete inhalation, close your left nostril and exhale from the right nostril. Then, inhale from the right nostril, and after complete inhalation close your right nostril and exhale from the left nostril. This is one round of Anuloma-Viloma Pranayama.

Repeat the procedure 5 times.

**for Pranav Mudra- fold the first two fingers of your right hand. Use your thumb to close your right nostril and ring finger to close your left nostril.*

12. Bhastrika Pranayama:

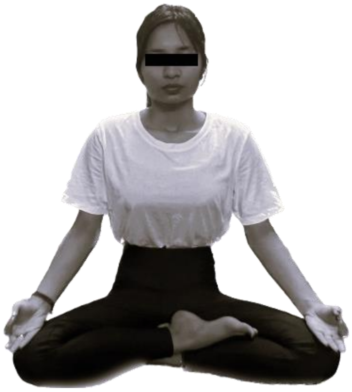

Forcefully inhale and forcefully exhale from both nostrils.

Breathe for 5 times in one round and repeat the practice for 5 times.

13. Bhramari Pranayama:

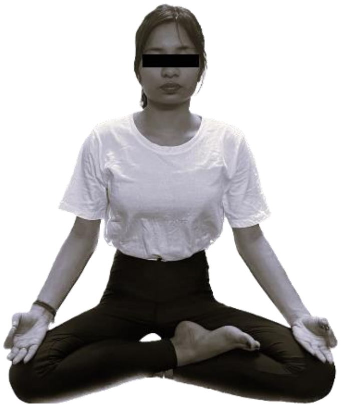

Inhale deeply and while exhaling produce a humming sound ('M' sound).

Repeat the practice 10 times.

14. Shavasana 0:

Lie down flatly on the ground for 2 minutes.

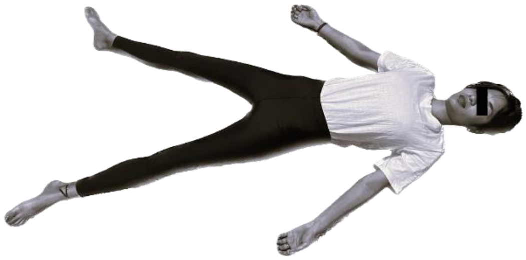

Shavasana 1:

Step 1: Lie down flatly on the ground. Keeping the right hand on the abdomen and left hand on the chest, observe your breath for 3 minutes.

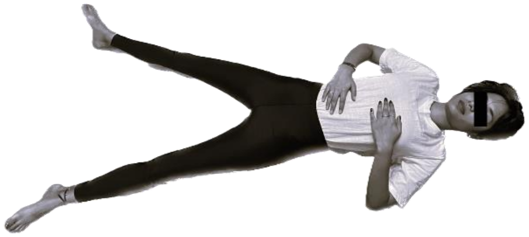

15. Soham Dhyana:

Sit in Swastikasana position.

While inhaling mentally chant ‘SO’ and while exhaling ‘HAM’.

Repeat this in mind for 10 minutes.

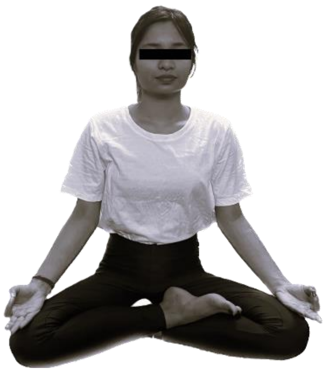

Supplement: Multimedia component 2 [file mmc2.pdf]
